# Integrin β1 Increases Stem Cell Survival and Cardiac Function after Myocardial Infarction

**DOI:** 10.3389/fphar.2017.00135

**Published:** 2017-03-17

**Authors:** Lili Li, Qifan Guan, Shuling Dai, Wen Wei, Yao Zhang

**Affiliations:** ^1^Department of Cardiology, The Second Affiliated Hospital, Harbin Medical UniversityHarbin, China; ^2^Department of Cardiology, Yunnan Fuwai Cardiovascular Disease HospitalKunming City, China; ^3^Department of Cardiac Rehabilitation, Shanxi Cardiovascular HospitalTaiyuan City, China; ^4^Department of Paediatrics, The First Affiliated Hospital, Harbin Medical UniversityHarbin, China

**Keywords:** integrin β1, bone mesenchymal stem cell, apoptosis, myocardial infarction, adhesion

## Abstract

Bone mesenchymal stem cells (BMSCs) transplantation is a promising therapeutic approach for myocardial infarction (MI), but its application is limited by poor viability of BMSCs. In this study, we aimed to improve the survival of BMSCs by lentivirus vector mediated overexpression of integrin β1. *In vitro* study showed that integrin β1 overexpression could facilitate the proliferation of BMSCs under oxygen glucose deprivation condition and regulated the expression of Caspase-3, Bax, Bcl-2, FAK, and ILK in BMSCs. Next, MI was induced in rat model and Igtb1BMSCs, NullBMSCs, or NatBMSCs were transplanted by intramyocardial injection. One week later, the survival of BMSCs was higher in Itgb1 BMSCs group than in other groups. Four weeks after transplantation, heart function was significantly improved in Igtb1BMSCs group compared to other groups. The expression levels of Caspase-3 and Bax were decreased while the expression levels of Bcl-2, FAK, ILK, and VEGF were increased in the cardiomyocytes of Igtb1BMSCs group compared to other groups. In conclusion, integrin β1 overexpression could increase the survival of BMSCs and improve the efficacy of transplanted BMSCs for MI treatment. The beneficial effects may be mediated by inhibiting the apoptosis of both transplanted BMSCs and cardiomyocytes through adhesion-mediated cell survival signaling.

## Introduction

Myocardial infarction is the most common cardiovascular disease threatening human health. Despite major advances in percutaneous coronary intervention, many patients of myocardial infarction still suffer from heart failure (Mielniczuk et al., 2007). Bone mesenchymal stem cells (BMSCs) transplantation, which can stimulate angiogenesis and/or myogenesis and attenuate ventricular remodeling, is a promising but by no means completely worldwide accepted and confirmed therapeutic approach for myocardial infarction (Bartunek et al., 2016; Du et al., 2016; Faiella and Atoui, 2016). However, poor viability of the transplanted cells limits the therapeutic effect. Some mechanisms have been proposed to explain the death of the transplanted cells. Anoikis is a programmed cell death induced by loss of matrix attachment and plays an important role in the death of the transplanted cells (Robey et al., 2008; Shi and Li, 2008; Amiri et al., 2015). To avoid anoikis and improve the survival of implanted BMSCs, it is important to promote the attachment of BMSCs to extracellular matrix (ECM).

Cell adhesion to ECM is mainly mediated by integrins. Integrins are implicated in cell-to-cell and cell-to-ECM adhesion by interacting with focal adhesion complex, which includes focal adhesion kinase (FAK), Src, talin, vinculin, paxillin, and integrin-linked kinase (ILK). Importantly, integrins regulate cell survival by interacting with focal adhesion complex and modulating the expression of apoptosis related genes (Michel, 2003; Zhan et al., 2004; Reddig and Juliano, 2005; Alanko et al., 2015; Cai et al., 2016). For example, integrins activate ILK and increase the phosphorylation of ERK and Akt. PI3K/Akt/ERK pathway is a well confirmed anti-apoptotic pathway (Heusch, 2015), which has been proven to be involved in the regulation of adhesion-mediated cell survival signaling in hypoxic BMSCs, and increase Bcl-2/Bax ratio to inhibit caspase-3 activation (Ding et al., 2009; Song et al., 2009; Mao et al., 2014). Therefore, we speculated that increasing integrin expression in BMSCs can improve their adhesion to ECM and inhibit the anoikis.

Furthermore, previous studies confirmed that integrin-mediated adhesion were preferentially mediated through integrin β1 and the deficiency of integrins β1 increased myocardial dysfunction and apoptosis after MI (Krishnamurthy et al., 2006; Takada et al., 2007). Therefore, in this study we constructed lentivirus vector containing gene for integrin β1 (Itgb1) and infected BMSCs with the recombinant virus to mediate overexpression of integrin β1 in BMSCs. Then we evaluated the effects on the survival of BMSCs *in vitro* and *in vivo* and the therapeutic effects of BMSCs transplantation in rat model of myocardial infarction.

## Methods

### Construction of lentivirus carrying Itgb1

Lentivirus vector containing ITGB1 (Len-Itgb1) was constructed by GeneChem (Shanghai, China) using the procedures reported previously (Wang K. et al., 2013). Briefly, rat Itgb1 cDNA (Accession No: NM_017022.2) was amplified by PCR with the following primers: forward, 5′- GAG GAT CCC CGG GTA CCG GTC GCC ACC ATG AAT TTG CAA CTG GTT TTC TG -3′; and reverse, 5′- TCA CCA TGG TGG CGA CCG5 GTT TTC CCT CAT ACT TCG GAT TG -3′. PCR conditions were pre-denaturation at 95°C for 3 min; 35 cycles of denaturation at 95°C for 12 s, annealing at 57°C for 45 s; and a final extension at 37°C for 1 min. The amplified sequence was digested with Age I and then inserted into GV218 lentiviral vector (GeneChem, Shanghai, China) to get Len-Itgb1. DNA sequencing was performed to verify the sequence of the insert and the identities were 100%. Western blot analysis was employed to confirm the overexpression of Itgb1 in transfected 293 T cells. Lentivirus without the insertion of Itgb1 (Len-null) but only expressing enhanced green fluorescent protein (EGFP) was routinely made as a control.

### Isolation and infection of BMSCs

BMSCs were isolated and passaged according to previously described methods (Li et al., 2008, 2009). Briefly, the femurs and tibias were removed from Wistar rats, and the bone marrow was flushed out using 10 ml Dulbecco's modified Eagle's medium/F12 (DMEM/F12; HyClone, Logan, UT, USA) with 1% penicillin/streptomycin (Beyotime Institute of Biotechnology, Nantong, China). After centrifugation, the cell pellets were resuspended in 6 ml DMEM/F12 supplemented with 10% fetal bovine serum (ScienCell Research Laboratories, San Diego, CA, USA) and 1% penicillin/streptomycin and plated in a 25 cm^2^ plastic flask at 37°C in a humidified atmosphere containing 5% CO_2_ and 95% air. Following culture for 48 h, the medium was changed and the non-adherent hematopoietic cells were removed. The spindle-shaped adherent cells were BMSCs. At the third passage, BMSCs were infected with Len-Itgb1 or Len-null. To select the best multiplicity of infection (MOI) for Lentivirus-mediated gene transfer, preliminary experiments were conducted according to the manufacturer's recommendations (GeneChem, Shanghai, China), and 1:20 was chosen as the optimal MOI because of high efficiency and low toxicity. BMSCs were divided into three groups, including infection with Len-ITGB1 (Itgb1-BMSCs), infection with Len-null (Null-BMSCs), and untreated native BMSCs (Nat-BMSCs). Seventy two hours after infection, BMSCs in all groups were treated with Oxygen glucose deprivation (OGD). Furthermore, integrin β1 expression in Itgb1-BMSCs, Null-BMSCs, and Nat-BMSCs was detected by RT-PCR and Western blot analysis.

### Oxygen glucose deprivation (OGD)

The procedures was performed as described previously (Wang L. et al., 2013). BMSCs in all groups were rinsed twice with serum-free, glucose-free, and sodium pyruvate-free DMEM and were cultured in the same medium at 37°C in an anoxia chamber saturated with 95% N_2_ and 5% CO_2_ for up to 12 h.

### Cell survival assay

Cell survival assay was performed with the use of the cell counting kit-8 according to the manufacturer's recommendations (Sigma). Briefly, BMSCs were cultured in 96-well culture plates at 1 × 10^4^ cells/well as monolayer. After lentiviral transduction and 12 h OGD stimulation, BMSCs in all groups were rinsed twice with PBS, then 10 μl of CCK-8 solution were added to each well. After incubation for 4 h, the cell proliferation was evaluated by measuring the absorbance at 450 nm using a microplate reader.

### TUNEL assay

Apoptosis of BMSCs was detected by terminal deoxynucleotidyl transferase-mediated-dUTP nick-end labeling (TUNEL) assay using the kit (Roche, Penzberg, Germany). Briefly, BMSCs were fixed in 4% paraformaldehyde, and then incubated with 3% H_2_O_2_ and 0.1% Triton X-100 in 0.1% sodium citrate. After washing in PBS, the cells were incubated with TUNEL reaction mixture at 37°C for 1 h in the dark. The cells were also incubated with 4-Diamino-2-phenylindole (DAPI, Beyotime Institue of Biotechnology) for 15 min. The cells were observed under a fluorescence microscope, and TUNEL^+^ and DAPI^+^ cells from 10 randomly selected fields were counted. Apoptotic index (AI) was calculated as: AI = (the number of TUNEL^+^ cells/total number of cells) × 100%.

### Reverse transcription polymerase chain reaction (RT-PCR)

Total RNA was extracted from different groups of BMSCs using Trizol reagent (Tiangen), and cDNA was synthesized with an AccuPower RocketScriptTM RT PreMix Kit (Bioneer). RT-PCR was performed using AccuPower Plus DualStarTM qPCR PreMix Kit (Bioneer) in a Bio-Rad iQ5 optical module. The primer sequences were as follows: Itgb1 (Accession No: NM_017022.2) forward 5′-AAAATGGACGAAAGTGCTCTAAC -3′, reverse 5′-TGGGACTTGCTGGGATGC -3′ (product size 587 bp); β-actin (Accession No: NM_031144.3) forward 5′-CGTAAAGACCTCTATGCCAACA -3′, reverse 5′-GGAGGAGCAATGATCTTGATCT -3′ (product size 398 bp). The PCR conditions were set for pre-denaturation at 95°C for 3 min; 35 cycles of denaturation at 95°C for 12 s, annealing at 58°C for 40 s; and a final extension at 37°C for 1 min. PCR products were size-fractionated by 1% agarose gel electrophoresis.

### Western blot analysis

Protein extracts (20 μg) from the different groups of BMSCs were loaded onto 6–12% SDS-PAGE gel (Beyotime), then subjected to electrophoresis for 90 min at 120 V and transferred to PVDF membranes. The membranes were blocked and incubated with primary antibodies overnight at 4°C, followed by washing and incubation with horseradish peroxidase (HRP)-conjugated secondary antibodies. Immunoreactive bands were visualized by enhanced chemiluminescence (ECL, Beyotime) and exposed to x-ray film. Relative protein expression was determined by image analysis using an Alpha Innotech gel analysis system.

The primary antibodies used were anti-Itgb1 (Abcam, USA, 1:500), anti-FAK (Abcam, USA, 1:1000), anti-ILK (Abcam, USA 1:5000), anti-VEGF (Abcam, USA 1:1000), anti-Bax (Cell Signaling Technology, USA 1:1000), anti-caspase 3 (Cell Signaling Technology, USA 1:1000), anti-Bcl-2 (Cell Signaling Technology, USA 1:1000), anti-β-actin (ZSGB BIO). The secondary antibodies were HRP-conjugated goat anti rabbit IgG (BIOSS, China 1:5000).

### Animal experiments

All animal procedures were approved by an institutional animal care and use committee and were performed according to the Guideline of the Ethical Committee of Harbin Medical University.

Myocardial infarction was established in male Wistar rats (200–220 g) according to previously described methods (Müller-Ehmsen et al., 2002; Dai et al., 2005). Briefly, rats were anesthetized with chloral hydrate. After the oral endotracheal intubation, animals were mechanically ventilated with room air using a small animal ventilator (rate 75 cycles/min, tidal volume 3 mL per 100 g of body weight, Harvard Apparatus Rodent Ventilator, model 683). ECG was monitored throughout the surgical procedure. The heart was exposed through a left thoracotomy, and the left anterior descending coronary artery (LAD) was ligated with a 5-0 polyester suture. After the observation of elevated ST segment, three groups of BMSCs (2 × 10^6^ cells in 100 μl DMEM) were respectively injected into the periphery region of infarcted myocardium. Control animals underwent LAD ligation and was injected with DMEM only. Successful injection was shown by the forming of a bleb covering the infarct zone.

### Assessment of cardiac function

Heart function was assessed by transthoracic echocardiography 4 weeks after MI using iE33 ultrasound system (Phillips) with a 15 MHz probe, because echocardiography is a more sensitive technique than invasive hemodynamics to evaluate left ventricle systolic function and cardiac damage (Ishikawa et al., 2012). Left ventricular (LV) parameters were obtained from two-dimensional images together with M-mode interrogation in long-axis view. Parameters measured included LV ejection fraction (LVEF), LV fractional shortening (LVFS), LV end-diastolic dimension (LVDd), LV end-systolic dimension (LVDs). All echocardiographic measurements were averaged from at least three separate cardiac cycles.

### Survival of transplanted BMSCs

To detect cell survival rate after BMSCs implantation, prior to transplantation, transfected BMSCs were fluorescently labeled with 4′,6-diamidine-2′-phenylindole dihydrochloride (DAPI) following the manufacturer's instructions. One week after transplantation, 8 rats (4 in Itgb1BMSCs group and 4 in NullBMSCs group) were euthanatized. The hearts were harvested and embedded in optimum cutting temperature (OCT) compound, snap-frozen in liquid nitrogen, and cut into sections. Transplanted cells which expressed green and blue fluorescence were observed by fluorescence microscopy in which six fields were randomly selected. The GFP+ and DAPI+ cells were counted manually.

### Immunohistochemical analysis

All rats were sacrificed 4 weeks after implantation, and their hearts were dissected. The cardiac tissues surrounding the infarcted zone were obtained for immunohistochemical analysis. Briefly, after cutting off the excess tissue and right ventricle, the hearts were perfusion-fixed with 10% (vol/vol) neutral buffered formaldehyde for 24 h, and embedded in paraffin. Sections of 2 μm thickness were mounted on gelatin-coated glass slides. After deparaffinization and rehydration, the sections were incubated with primary antibodies against FAK (Abcam, USA, 1:150), caspase-3 (Cell Signaling Technology, USA 1:200), and β-actin (ZSGB BIO, China, 1:300), followed by incubation with HRP-conjugated goat anti rabbit IgG (BOSTER, Wuhan, China). Six randomly selected fields per section were analyzed and scanned with a digital image analyzer, the expression of FAK and caspase-3 was calculated as the sum of all brown staining areas divided by the total area of the image.

### Statistical analysis

Experiments were performed in quadruplicate and repeated at least three times. All data were expressed as mean + SEM. Student's *t*-test or one-way ANOVA was performed with GraphPad Prism 5 to analyze the differences. *P* < 0.05 was considered statistically significant.

## Results

### Overexpression of integrin β1 in BMSCs

**Figure 1:**
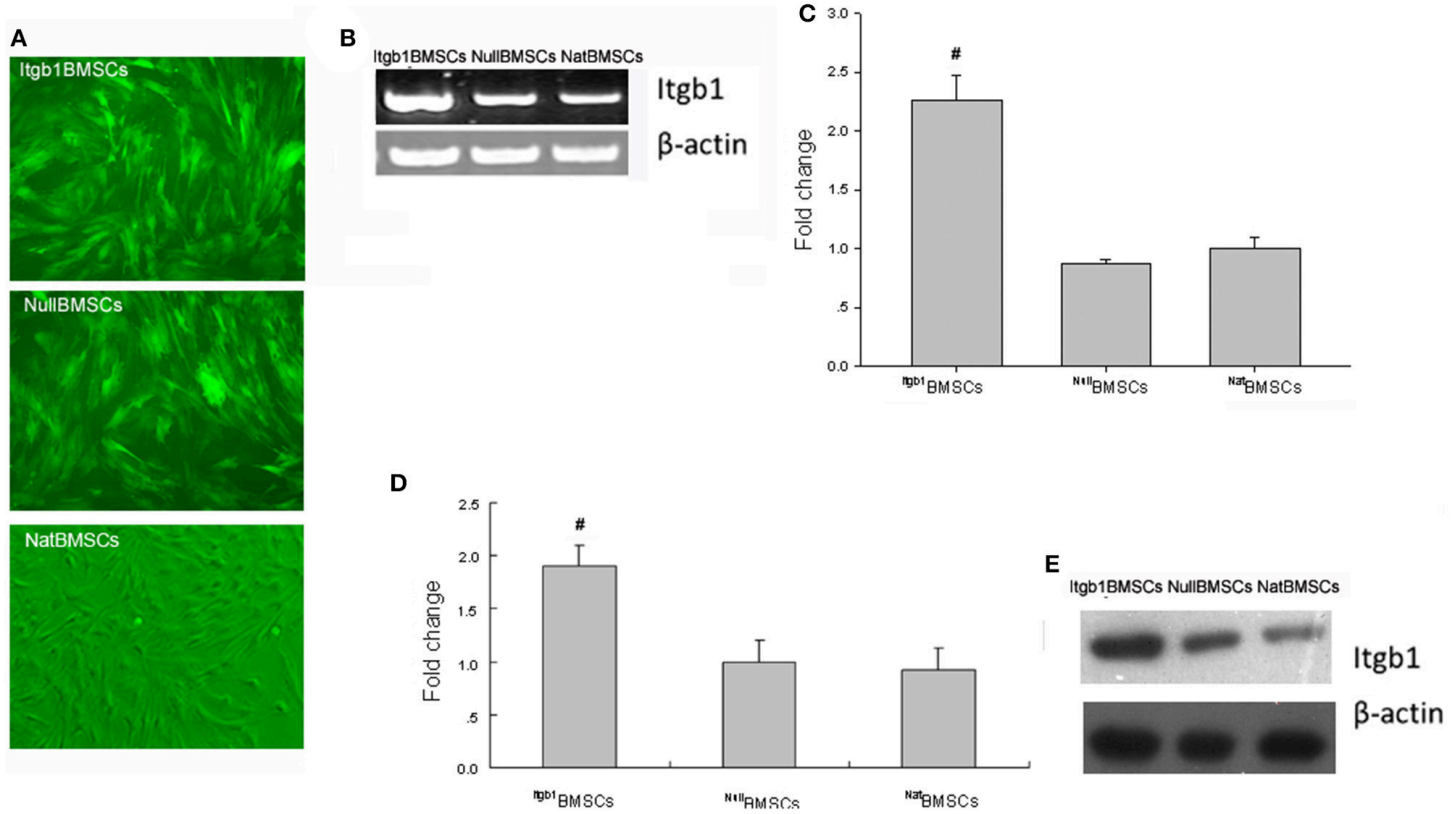
**Expression of integrin β1 in BMSCs. (A)** BMSCs were observed under fluorescent microscope (MOI = 1:20); **(B,C)**. RT-PCR analysis of integrin β1 mRNA expression. **(D,E)**. Western blot analysis of integrin β1 expression. Fold change of integrin β1 level was calculated after normalization to ^Nat^BMSCs (set as 1). #*p* < 0.01 vs. ^Nat^BMSCs and ^Null^BMSCs.

As shown in Figure 1A, most of the infected cells showed green fluorescence under fluorescence microscopy. RT-PCR showed that the expression of Itgb1 mRNA was significantly higher in Itgb1BMSCs than NatBMSCs and NullBMSCs (Figures 1B,C). Western blot analysis showed that the expression of Itgb1 protein was significantly higher in Itgb1BMSCs (Figures 1D,E).

### Integrin β1 overexpression promoted BMSCs survival under OGD

**Figure 2:**
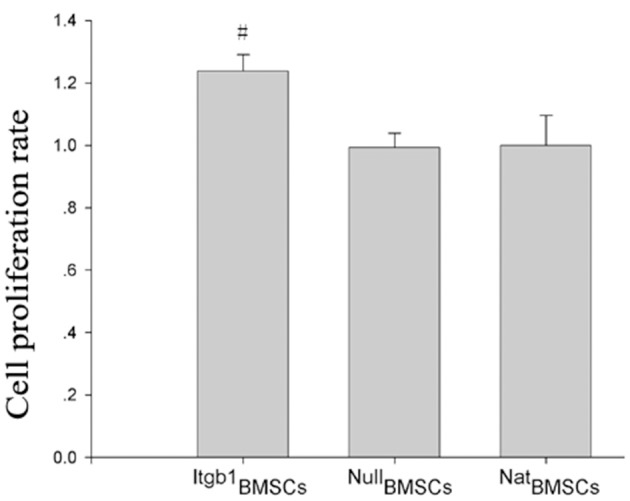
**The survival of BMSCs under oxygen glucose deprivation**. Cell survival was analyzed by CCK8 assay. #*p* < 0.05 vs. ^Nat^BMSCs and ^Null^BMSCs.

**Figure 3:**
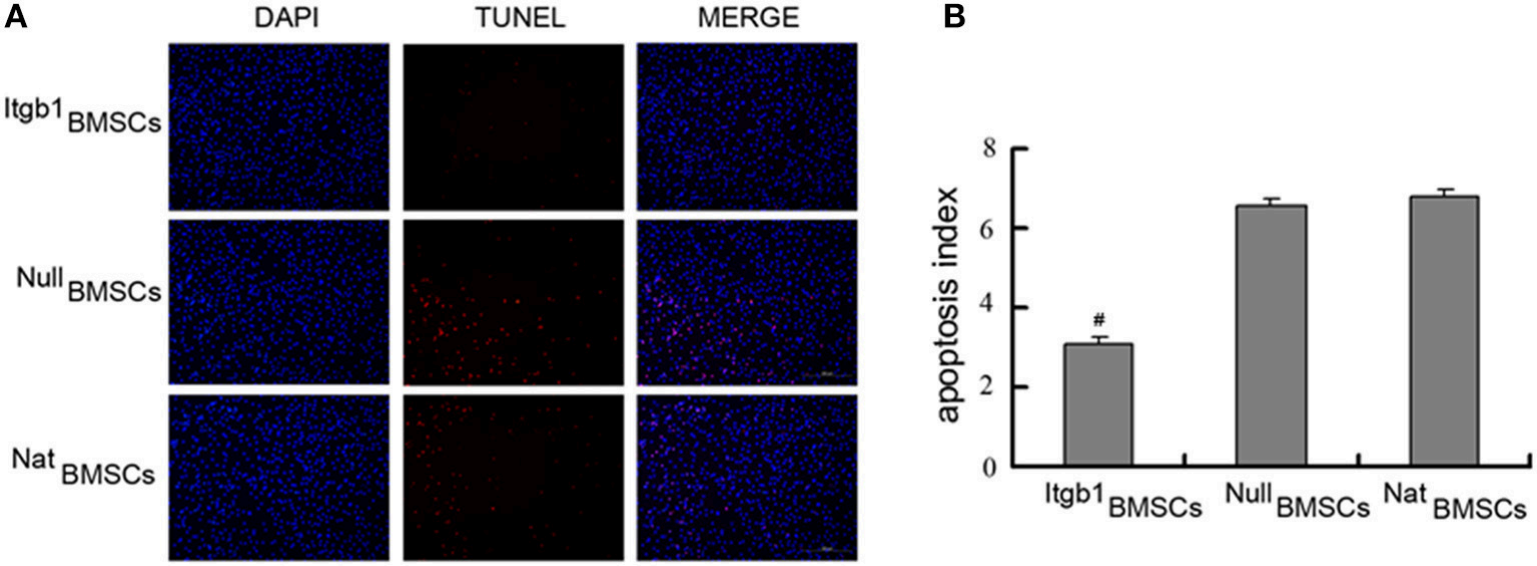
**The apoptosis of BMSCs under oxygen glucose deprivation. (A)** Representative TUNEL staining of BMSCs. Apoptotic cells had red staining in the nuclei, while all cells were stained blue by DAPI. **(B)** Quantitative analysis of TUNEL staining. #*p* < 0.01 vs. ^Nat^BMSCs and ^Null^BMSCs.

To investigate the effect of Integrin β1 overexpression on the survival of BMSCs, we performed CCK8 assay and TUNEL assay. CCK8 assay showed that the survival rate of Itgb1BMSCs was significantly higher than that of Nat BMSCs and NullBMSCs under OGD condition (Figure 2). Furthermore, TUNEL assay showed that the apoptotic index was significantly decreased in Itgb1BMSCs compared with NatBMSCs and NullBMSCs (Figure 3).

### Integrin β1 overexpression inhibited the apoptosis and facilitated the adhesion of BMSCs

**Figure 4:**
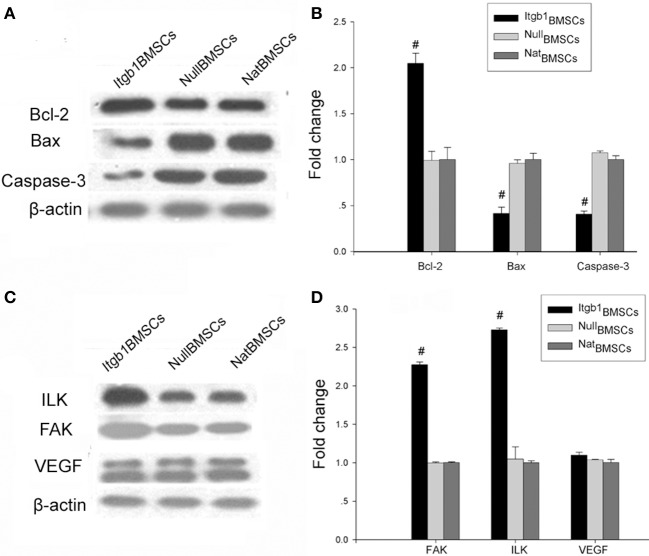
**The expression of apoptosis and adhesion related proteins in BMSCs under oxygen glucose deprivation. (A)** Western blot analysis of apoptosis related proteins. β-actin was loading control. **(B)** Densitometry analysis of fold change of protein levels. Protein levels in ^Nat^BMSCs were set as 1. **(C)** Western blot analysis of adhesion related proteins. β-actin was loading control. **(D)** Densitometry analysis of fold change of protein levels. Protein levels in ^Nat^BMSCs were set as 1. #*p* < 0.01 vs. ^Nat^BMSCs.

We then investigated the effect of Integrin β1 overexpression on the survival and adhesion related proteins in BMSCs. Western blot analysis showed that compared with NatBMSCs and Null BMSCs, anti-apoptotic protein Bcl-2 was significantly increased while Caspase-3 and pro-apoptotic protein Bax were decreased in Itgb1BMSCs group (Figures 4A,B). Moreover, cell adhesion related proteins FAK and ILK were significantly increased in Itgb1BMSCs group (*P* < 0.01, Figures 4C,D). Because hypoxia is an important factor leading to the apoptosis of transplanted MSCs, we investigated whether integrin β1 overexpression could increase the ability of BMSCs to express VEGF, which may lead to the formation of capillary and increase the supply of nutrient and oxygen to BMSCs. However, we found that the expression of VEGF showed no significant difference among the three groups (Figures 4C,D).

### Itgb1BMSCs transplantation improved rat cardiac function

**Figure 5:**
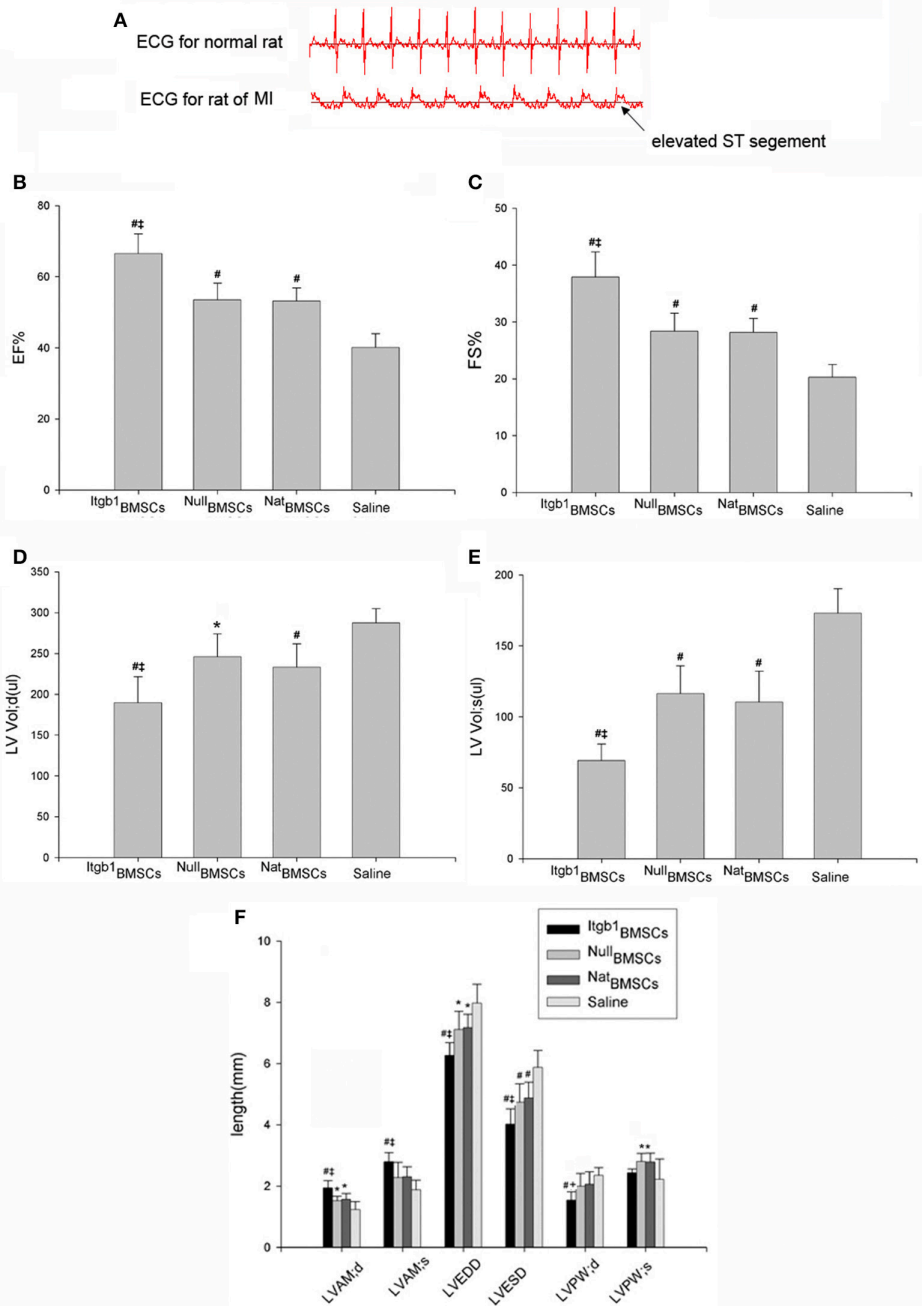
**Integrin β1 overexpression improved cardiac function in rat model of MI. (A)** ECG showed elevated ST segment in rat model of MI. **(B–F)** Echocardiographic assessment of cardiac function in four groups of rats. ^Itgb1^BMSCs group, ^Null^BMSCs group, ^Nat^BMSCs group, and Saline group were injected into the periphery region of infarcted myocardium with ^Itgb1^BMSCs, ^Null^BMSCs, ^Nat^BMSCs, and saline, respectively. #*p* < 0.01 vs. Saline group; ^*^p < 0.05 vs. Saline group; ‡*p* < 0.01 vs. ^Nat^BMSCs group.

MI model was successfully established in 27 rats based on the results of ECG (Figure 5A). The 27 rats were randomly divided into four groups (7 in Itgb1BMSCs group, 7 in NullBMSCs group, 7 in NatBMSCs group, and 6 in saline group), and then were injected with BMSCs or saline, respectively. Four weeks after transplantation, transthoracic echocardiography was conducted. The results showed that ejection fraction (EF) and fractional shortening (FS) were improved in NullMSCs group and NatMSCs group compared to saline group (Table 1, Figures 5B,C). Furthermore, the EF and FS were further increased in Itgb1MSCs group compared with NullMSCs group and NatMSCs group (Table 1, Figures 5B,C). In addition, compared to other three groups, left ventricular volume both in diastole and systole, left ventricular anterior wall (LVAW), LV end-diastolic dimension (LVEDD), and end-systolic dimension (LVESD) were significantly decreased in Itgb1MSCs group (Table 1, Figures 5D–F).

**Table 1:** **Echocardiographic assessment of cardiac function**.

	**EF (%)**		**FS (%)**		**LV Vol;d (**μ**l)**		**LV Vol;s (**μ**l)**	
^Itgb1^MSCs	66.50 ± 2.10^#^^‡^		37.88 ± 1.69^#^^‡^		189.6 ± 12.11^#^^‡^		69.07 ± 4.41^#^^‡^	
^Null^MSCs	53.49 ± 1.94^#^		28.36 ± 1.31^#^		246.0 ± 11.39^†^		116.4 ± 8.03^#^	
^Nat^MSCs	53.18 ± 1.49^#^		28.19 ± 0.99^#^		233.1 ± 11.66^#^		110.4 ± 8.91^#^	
Control	40.11 ± 1.73		20.26 ± 1.00		287.4 ± 7.85		173.1 ± 7.76	
	LVAW;d (mm)	LVAW;s (mm)		LVEDD (mm)	LVESD (mm)	LVPW;d (mm)		LVPW;s (mm)
^Itgb1^MSCs	1.94 ± 0.09^#^^‡^	2.79 ± 0.11^#^^‡^		6.27 ± 0.16^#^^‡^	4.02 ± 0.19^#^^‡^	1.69 ± 0.10^#^^†^		2.43 ± 0.05
^Null^MSCs	1.52 ± 0.06^*^	2.28 ± 0.20		7.11 ± 0.24^*^	4.74 ± 0.24^#^	1.96 ± 0.15		2.65 ± 0.16^*^
^Nat^MSCs	1.57 ± 0.08^*^	2.31 ± 0.13		7.17 ± 0.18^*^	4.87 ± 0.21^#^	2.01 ± 0.13		2.52 ± 0.22^*^
Control	1.24 ± 0.12	1.88 ± 0.14		7.97 ± 0.32	5.87 ± 0.25	2.15 ± 0.12		2.22 ± 0.29

# p < 0.01 vs. Control group;

* *p < 0.05 vs. Control group*;

‡ *p < 0.01 vs. NatBMSCs group*;

† *p < 0.05 vs. NatBMSCs group*.

*Comparisons of parameters among groups were analyzed using ANOVA*.

### Integrin β1 overexpression improved the survival of transplanted BMSCs

**Figure 6:**
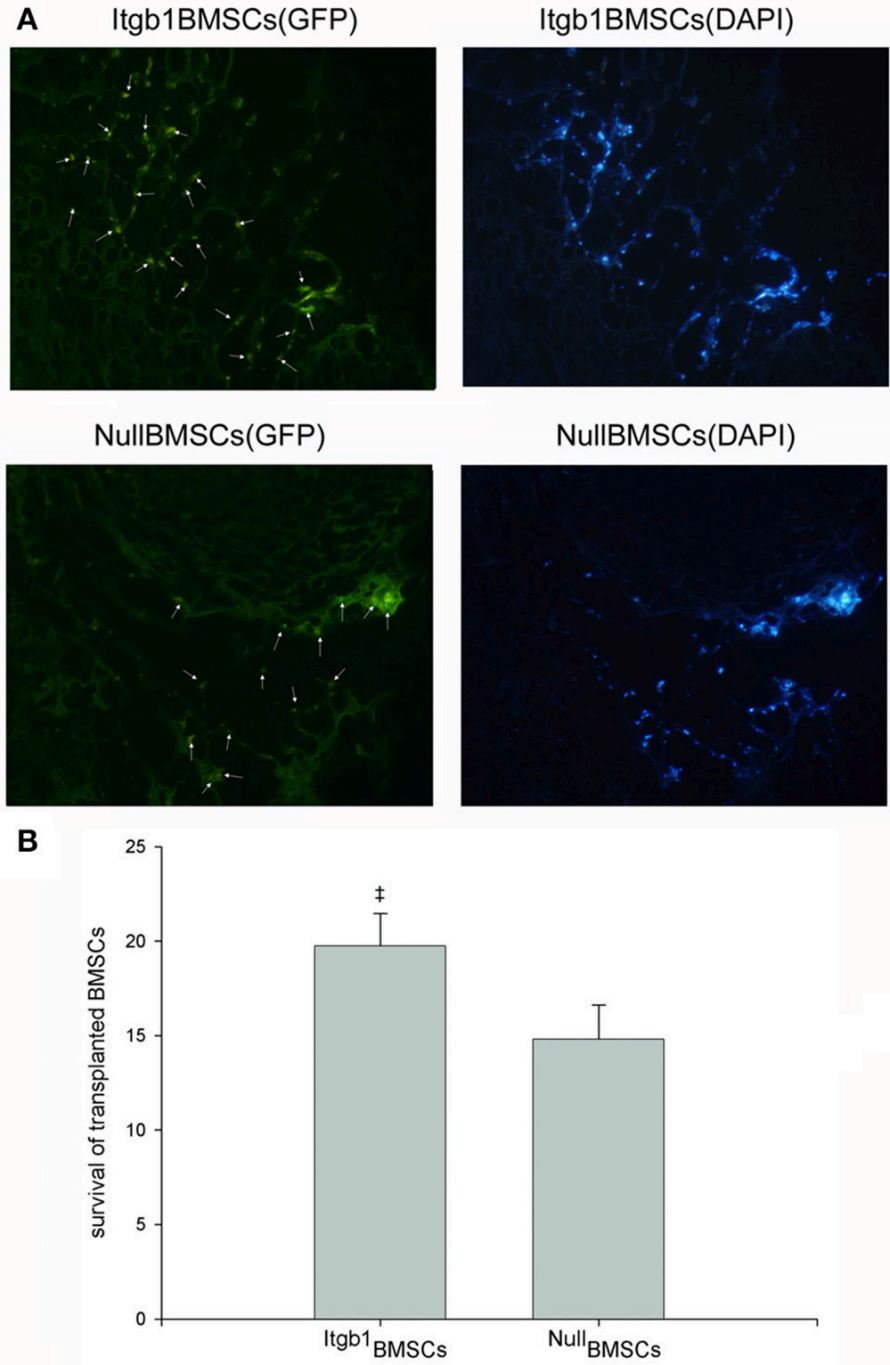
**Survival of transplanted BMSCs. (A)** Representative images showing significant increase of transplanted BMSCs in ^Itgb1^BMSCs group. Survived BMSCs were marked by the arrows and they were stained blue by DAPI. **(B)** Quantitative analysis of the number of survived BMSCs in each field. ‡*p* < 0.01 vs. ^Null^BMSCs group.

One week after transplantation, 8 rats (4 in Itgb1BMSCs group and 4 in NullBMSCs group) were euthanatized. The hearts were harvested to get transplanted BMSCs. The result showed that the transplanted Itgb1MSCs had a higher survival rate compared to transplanted NullBMSCs (Figure 6).

### Integrin β1 overexpression inhibited the apoptosis of myocardium

**Figure 7:**
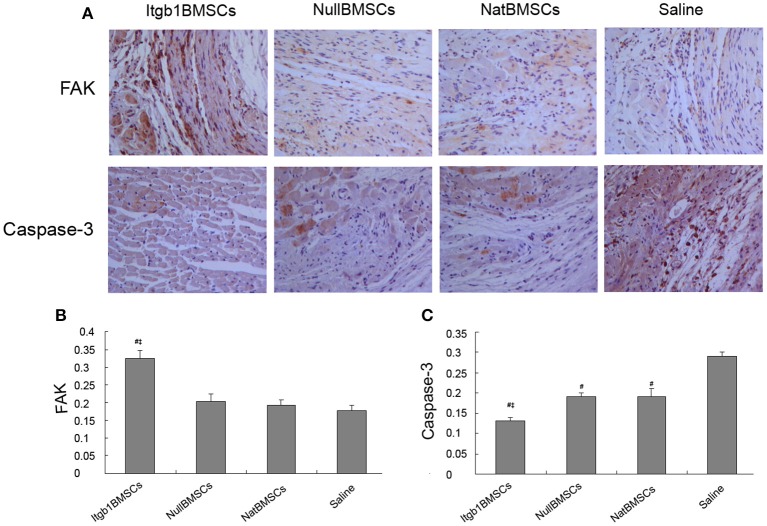
**Immunohistochemical analysis of FAK and Caspase-3 in the myocardium of different groups of rats. (A)** Representative immunohistochemical images. **(B,C)** Quantitative analysis of the expression of FAK and Caspase-3. #*p* < 0.01 vs. Saline group; ‡*p* < 0.01 vs. ^Nat^BMSCs group.

**Figure 8:**
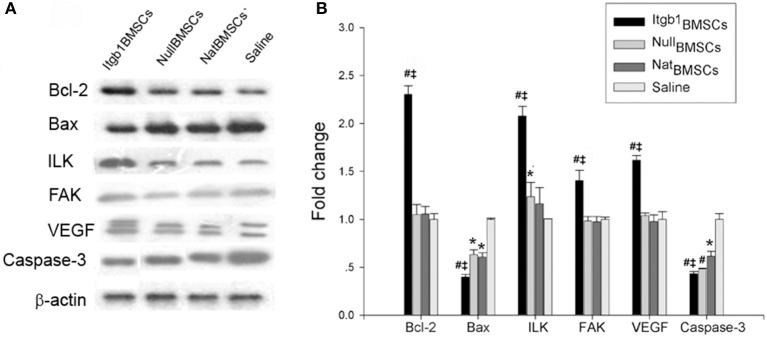
**Expression levels of apoptosis and adhesion related proteins in the myocardium of different groups of rats**. ^Itgb1^BMSCs group, ^Null^BMSCs group, ^Nat^BMSCs group, and Saline group were injected into the periphery region of infarcted myocardium with ^Itgb1^BMSCs, ^Null^BMSCs, ^Nat^BMSCs, and saline, respectively. **(A)** Western blot analysis of apoptosis and adhesion related proteins. β-actin was loading control. **(B)** Densitometry analysis of fold change of protein levels. #*p* < 0.01 vs. Saline group; ^*^p < 0.05 vs. Saline group; ‡*p* < 0.01 vs. ^Nat^BMSCs group.

Four weeks after transplantation, all rats were sacrificed and cardiac muscles were collected for immunohistochemical and Western blot analysis. Immunohistochemical analysis showed that compared with saline group, the expression of FAK was increased and the expression of caspase-3 was decreased in NullMSCs group and NatMSCs group and there was no difference between NullMSCs group and NatMSCs group. Furthermore, the expression of FAK was increased and the expression of caspase-3 was decreased in Itgb1MSCs group compare with NullMSCs group and NatMSCs group (Figure 7). Furthermore, Western blot analysis showed that the expression levels of FAK were increased in NullMSCs group and NatMSCs group, while pro-apoptotic proteins including caspase-3 and Bax were decreased in NullMSCs group and NatMSCs group, compared to saline group (Figure 8). There were not difference in the expression of Bcl-2, FAK, and VEGF among NullMSCs group, NatBMSCs group and saline group (Figure 8). Notably, the expression of Bcl-2, FAK, ILK, and VEGF further increased and the expression of caspase-3 and Bax further decreased in Itgb1MSCs group (Figure 8).

## Discussion

In the present study, for the first time we evaluated the efficacy of Itgb1BMSCs transplantation for MI in a rat model. Our results showed that integrin β1 overexpression could increase the adhesion and inhibit the apoptosis of BMSCs under hypoxia condition. In addition, *in vivo* data demonstrated that transplanted ITGB1-BMSCs could protect the cardiomyocytes in the rat model of MI.

BMSCs become an ideal choice to treat MI, owing to their self-renewal, multi-line differentiation potential, and low immunogenicity (Du et al., 2016; Faiella and Atoui, 2016). However, transplanted BMSCs are confronted with cell death within a few days after transplantation due to a combination of harsh environmental conditions, anoikis and inflammation. Once BMSCs are injected into damaged tissues or organs, they encounter nutrient and oxygen deprivation, inflammation and a lack of matrix support and adhesion to ECM, which promote apoptotic signaling. There are various strategies for strengthening the therapeutic potential of transplanted BMSCs, including pretreatment with growth factors or cytokines, preconditioning with hypoxia, and the use of genetic modification to overexpress protective factors (Haider and Ashraf, 2008; Robey et al., 2008). In this study, we tried to decrease BMSCs death by inhibiting anoikis induced by loss of matrix attachment.

Integrins play an important role in mediating cell-to-cell and cell-to-ECM adhesion and previous study has confirmed that integrin-mediated adhesion were preferentially mediated through integrin β1 (Takada et al., 2007). Current studies reported inconsistent results about the expression of integrin β1 in BMSCs under hypoxia (Song et al., 2010; Saller et al., 2012). In this study, we employed lentivirus vector to mediate the overexpression of integrin β1 in BMSCs. Our results showed that BMSCs overexpressing integrinβ1 showed higher survival ability under OGD, accompanied by significantly increased levels of FAK, ILK and Bcl-2 and decreased levels of Bax and Caspase-3. These data suggest that integrin β1 could inhibit cell apoptosis by activating FAK and ILK, which subsequently regulate adhesion-mediated cell survival signals such as Bcl-2, Bax and caspase-3.

It is well known that cardiomyocyte apoptosis contributes to the progression of MI and is a major determinant of unfavorable LV remodeling. Anti-apoptotic treatment at an early stage can decrease the infarct size (Santos-Gallego et al., 2016). Previous study has demonstrated that the deficiency of integrins β1 increased myocardial dysfunction and apoptosis after MI (Krishnamurthy et al., 2006). In this study, we found that Itgb1BMSCs transplantation could decrease the expression of pro-apoptotic proteins caspase-3 and bax and increase the expression of anti-apoptic protein such as bcl-2 in the myocardium. Furthermore, the expression of ILK and FAK increased in Itgb1BMSCs group. Integrin β1 is involved in the anchorage of cardiomyocytes to ECM (Michel, 2003). Therefore, we speculate that transplanted Itgb1BMSCs could protect cardiomyocytes by inhibiting cardiomyocyte apoptosis in MI via increasing the expression of adhesion related molecules such as ILK and FAK, which subsequently regulate the expression of adhesion mediated cell survival proteins such as Bcl-2, Bax, and caspase-3.

Furthermore, our results shown that compared with saline, NullBMSCs and NatBMSCs transplantation could improve heart function, which support that the paracrine mechanism play an important role in BMSCs transplantation. BMSCs can secreted many cytokines such as VEGF. In addition, nutrient and oxygen deprivation is an important factor leading to the apoptosis of cardiomyocytes and transplanted BMSCs. In this study, the expression of VEGF was investigated in BMSCs and myocardium. Our results showed no significant difference in VEGF expression in Itgb1BMSCs compared to NullBMSCs and NatBMSCs, but the expression of VEGF was significantly increased in the myocardium in Itgb1BMSCs group. Thus, we conclude that integrin β1 overexpression can not influence VEGF secretion of individual BMSCs, but can increase VEGF expression in myocardium by inhibiting the apoptosis of transplanted BMSCs. Improved expression of VEGF is another mechanism to improve the survival of cardiomyocytes.

Our study has several limitations. First, the detailed molecular mechanism by which integrin β1 promotes the survival of transplanted BMSCs is not further addressed in this study. Second, our study is based on MI rat model, and our results should be confirmed in additional animal models of MI.

## Conclusion

We genetically engineered BMSCs to overexpress integrin β1 to increase the survival and efficacy of transplanted BMSCs in MI rat model. The beneficial effect of Itgb1BMSCs transplantation may be mediated by inhibiting the apoptosis of transplanted BMSCs and cardiomyocytes due to adhesion-mediated cell survival signaling. Further studies are needed to understand the detailed mechanism of adhesion related signaling in BMSCs to develop engrafted stem cell therapy for cardiac repair in ischemic-injured hearts.

## Author contributions

LL, QG, and SD performed the experiments, WW analyzed the data, YZ designed the study and wrote the manuscript.

### Conflict of interest statement

The authors declare that the research was conducted in the absence of any commercial or financial relationships that could be construed as a potential conflict of interest.
